# Assessing Suitable Habitats for *Gerbera piloselloides* (L.)Cass. in China Using an Optimized MaxEnt Model and Key Environmental Drivers

**DOI:** 10.3390/biology14070769

**Published:** 2025-06-26

**Authors:** Juan Xue, Longjiang Liu, Yan Li, Yan Zhang, Shanshan Liang, Huifang Chai

**Affiliations:** School of Pharmacy, Guizhou University of Traditional Chinese Medicine, Guiyang 550025, China; xuejuan062@gzy.edu.cn (J.X.); liulongjiang106@gzy.edu.cn (L.L.); 18753851087@163.com (Y.L.); zhangyan0003@gzy.edu.cn (Y.Z.)

**Keywords:** *Gerbera piloselloides* (L.)Cass., MaxEnt model, suitable habitats, environmental factors, artificial cultivation

## Abstract

*Gerbera piloselloides* (L.)Cass. is an important ethnomedicinal plant. Wild populations are steadily declining, however, posing a risk of failure to meet future market demand. Investigating suitable habitats for this plant and key influencing factors is crucial for its artificial cultivation. In this study, we implemented an optimized MaxEnt to project suitable habitats for the target species in five different periods and explored the impact of different environmental variables on its distribution. Our study findings provide confirmation of the following: (1) The current and future potential distributions of *Gerbera piloselloides* are primarily located in the areas south of the Qinling–Huaihe boundary in China, with future expansions expected to shift northward and westward. (2) Notably, there was a contraction in the junction regions of the provinces of Jiangxi, Anhui, Hunan, and Hubei. (3) The number of highly suitable habitats in Fujian, Guangdong, Yunnan, and Guangxi is expanding; in comparison, the number in Guizhou, Sichuan, and Hainan is declining. (4) The primary environmental determinants shaping its range, together with the threshold values for highly suitable habitats, are the minimum temperature of the coldest month (bio6, 0.88–22.58 °C) and the temperature seasonality (bio4, 461.54–763.9). The findings presented in this work provide support for the artificial cultivation and conservation of this plant.

## Simple Summary

*Gerbera piloselloides*, a medicinal plant used in cough remedies and local cuisine, faces declining wild populations due to overharvesting and climate change. In this study, we mapped the plant’s optimal current and future (through 2100) growth zones to provide data to support conservation and cultivation efforts. Our computer modeling results demonstrate that the species thrives in southern China, where winter temperatures remain mild (0.88 to 22.58 °C) with stable seasonal variations. While its habitat may expand northward and westward later in the century, some areas such as the Yangtze River Basin could become flood-prone. Yunnan province and Guangxi Zhuang Autonomous Region (Guangxi) are projected to become increasingly suitable areas for cultivation. These findings will aid in guiding sustainable farming and conservation strategies to protect this valuable species.

## 1. Introduction

Global mean temperatures have climbed notably over the past century, with the rate of warming exhibiting particular acceleration in recent decades [1]. Climate change can alter temperature, precipitation, and other conditions in the primary habitats of medicinal plants, thereby impacting their distribution [2]. Furthermore, different species respond to climate change in varying manners, potentially exhibiting either positive or negative reactions, which necessitates further analysis of the impacts of these climatic factors on such species [3]. Comprehending climate change effects on plant distribution dynamics and determining pivotal climatic constraints are therefore essential for species conservation and sustainable utilization.

Ecological niche modeling employs bioclimatic projections to forecast spatiotemporal shifts in species’ potential ranges and analyze bioclimatic constraints on species distributions [4]. Theoretically, these models can predict the global distribution of species and have the potential to guide the introduction of species to suitable regions [5]. At present, various computational models are utilized to predict potential suitable habitats for different species, including MaxEnt (maximum entropy), GARP (Genetic Algorithm for Rule-set Production), BIOCLIM (bioclimatic modeling), and Domain (environmental envelope) [6]. Among existing approaches, MaxEnt stands out as a frequently utilized tool for analyzing species distributions. It is renowned for its efficiency and ability to produce reliable results based solely on presence data [7,8].

**Figure 1:**
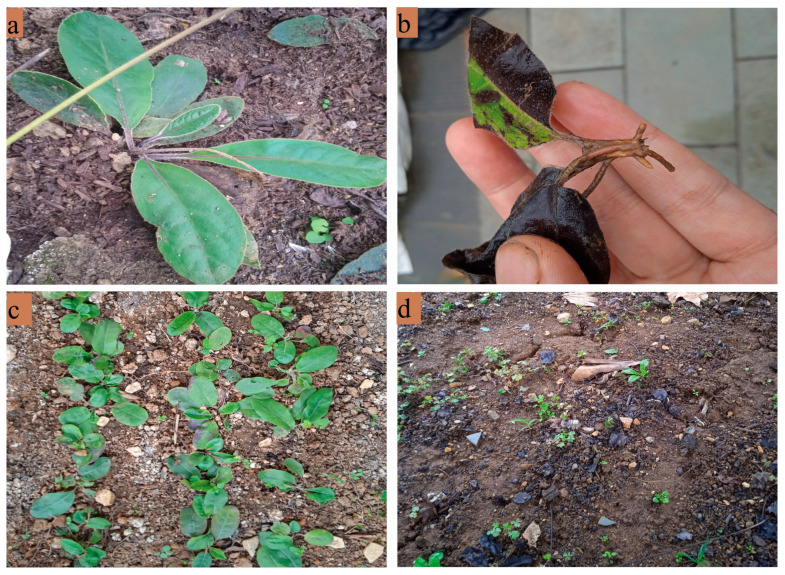
*Gerbera piloselloides*: (**a**) In the germplasm nursery; (**b**) *Gerbera piloselloides* affected by disease during winter; (**c**) healthy *Gerbera piloselloides* before disease/infection; (**d**) *Gerbera piloselloides* post disease.

As a species in the Asteraceae family, *Gerbera piloselloides* (L.)Cass. falls within the *Gerbera* genus; also known as Baiwei or White-browed grass, it grows on forest edges, grasslands, or wastelands and is primarily found in Guizhou, Yunnan, Guangxi, and other regions of China [9,10]. *Gerbera piloselloides* exhibits therapeutic effects by promoting lung cough, inducing sweating and promoting diuresis, and improving qi and blood circulation. It is often used in the treatment of cough, asthma, lung carbuncles, and other diseases [11]. Xingpi Yang’er Granules, developed jointly by the School of Pharmacy at Rutgers, The State University of New Jersey (USA), China Pharmaceutical University, and Shenyang Pharmaceutical University using *Gerbera piloselloides* as a raw material, have been shown to aid digestion, strengthen the intestines to relieve diarrhea, nourish blood, calm the mind, and relieve spasms; they are used to treat various pediatric conditions such as anorexia, diarrhea, night sweats, and enuresis [12,13]. Beyond its therapeutic value, *Gerbera piloselloides* serves dual purposes in Southwest China’s ethnic communities, functioning both as a component of traditional fermentation starters (jiuqu) and a culinary seasoning for meat dishes [14]. Despite its broad geographical distribution, the species’ medicinal applications necessitate whole-plant harvesting, a practice still largely dependent on wild collection. The increasing demand for its application in medicine and other fields has resulted in the significant depletion of natural populations in certain geographical areas. To protect this valuable plant resource and meet market demand, research on artificial cultivation has become an urgent priority. However, during artificial cultivation, it has been observed that the roots of *Gerbera piloselloides* rot in winter, while the leaves blacken and wilt, and even the entire plant dies, significantly reducing cultivation success rates (Figure 1). At present, researchers investigating *Gerbera piloselloides* primarily focus on its chemical composition [15], pharmacological properties [16], extraction techniques [17], quality standards [18], and proprietary Chinese medicines [19]. However, the possible distribution range of *Gerbera piloselloides* and the limiting factors remain unclear. This knowledge gap hinders research on its artificial cultivation. Consequently, there is an urgent need to systematically investigate *Gerbera piloselloides*’ potential distribution in order to delineate its suitable habitats and environmental constraints. Considering accelerating climate change impacts, assessing its distributional responses becomes equally crucial. These findings will offer theoretical foundations and practical guidelines for cultivated production.

**Figure 2:**
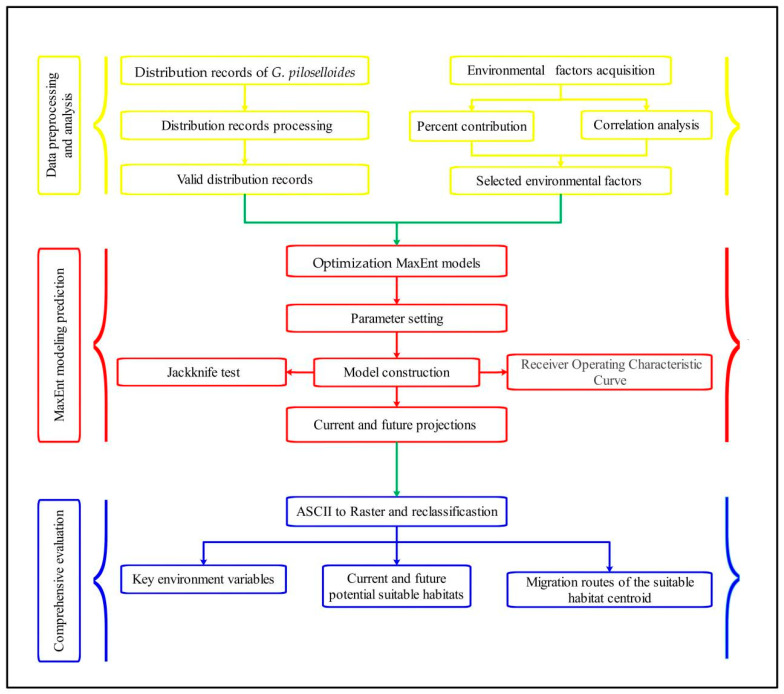
Framework of the research process.

Our study objectives were as follows: (1) mapping the current suitable range; (2) projecting future distributions under climate change scenarios; (3) identifying key limiting environmental factors; and (4) evaluating optimal introduction zones and conservation priority areas based on habitat suitability thresholds, with the research process illustrated in Figure 2.

## 2. Materials and Methods

### 2.1. Gathering and Analysis of Distribution Records

**Figure 3:**
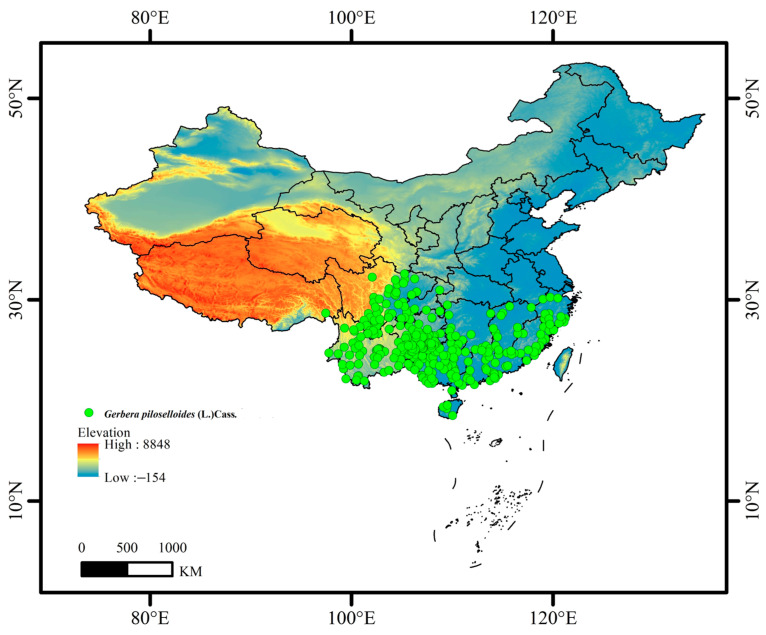
Valid distribution records of *Gerbera piloselloides* in China.

Distribution records of *Gerbera piloselloides* were obtained from the Chinese Virtual Herbarium (CVH, https://www.cvh.ac.cn/, accessed on 16 November 2024), the Plant Photo Bank of China (PPBC, https://ppbc.iplant.cn/, accessed on 18 November 2024), and the National Specimen Information Infrastructure (NSII, http://www.nsii.org.cn/, accessed on 19 November 2024). Records containing errors, duplicates, or incomplete data were removed. When encountering incomplete records (containing only city-level information), we implemented a three-step verification protocol: (1) categorization into precise (with detailed descriptions) or city-level records; (2) validation of city-level records by reviewing related literature and media reports; and (3) exclusion of records unverifiable through these methods. The geographic coordinates (latitude and longitude) for the species distribution records were obtained from Baidu Maps. To avoid overfitting and align with the 2.5 min resolution (5 × 5 km) of environmental data, only one randomly selected occurrence record per grid cell was retained [20]. Following a series of screening and processing steps, 334 valid distribution records were ultimately obtained. The distribution records were saved in a CSV format file (Figure 3).

### 2.2. Environmental Factors’ Acquisition and Selection

Five time periods were considered in the present study as follows: the current period (1970–2000) and four future projection windows (2030s: 2021–2040; 2050s: 2041–2060; 2070s: 2061–2080; 2090s: 2081–2100). The environmental dataset incorporated 58 variables, comprising 22 climatic and topographic parameters from WorldClim (19 bioclimatic and 3 topographic) combined with 36 edaphic factors from the World Soil Database (see Table S1 for complete specifications). Future climate projections were derived from the BCC-CSM2-MR model (CMIP6), incorporating both SSP126 (low emissions) and SSP585 (high emissions) scenarios across four time periods. All environmental factor layers were standardized to the same extent, using the WGS1984 geographic coordinate system with a resolution of 2.5 min. In this study, we presumed that all non-climatic variables would remain unchanged over the coming decades [21].

To prevent possible overfitting due to environmental factors, initial MaxEnt modeling was performed using the current 58 environmental factors to determine the percent contribution of these factors. Only environmental factors with a percent contribution of ≥1% were retained. Subsequently, ArcGIS 10.8 was employed to derive environmental factors at all distribution points, followed by correlation analysis in SPSS 26.0. For environmental factors exhibiting a strong correlation (|r| > 0.8), the variable making the lesser contribution to model performance was removed [22]. This process yielded 8 final variables for modeling (Table 1).

### 2.3. Optimization Models

Parameter optimization was conducted via the ENMeval R package version 4.3.2, systematically evaluating regularization multipliers (0.5–4 in 0.5 increments) and five feature class types (L, T, Q, H, and P) across 48 possible combinations. The final model configuration was selected based on minimal AICc scores (delta.AICc = 0) to balance model fit and complexity [23,24].

### 2.4. Model Building and Accuracy Assessment

The distribution records of *Gerbera piloselloides* and environmental factors were incorporated into MaxEnt modeling software version 3.4.3. The model parameters were set as follows: optimized RM and FC parameters were chosen, with 25% of the distribution data used as the test set and 75% used as the training set. The replication type was set to bootstrap, with 10 replicates. It was determined that the output format would be Logistic, and the other configurations were kept as default. Model performance was evaluated using the area under the curve (AUC) of the receiver operating characteristic (ROC), spanning 0–1, with values approaching 1 signifying superior predictive performance [25].

### 2.5. Data Processing

The output results (ASCII format files) were transformed into raster format utilizing ArcGIS software. Four habitat suitability classes were derived by applying natural breaks classification to the occurrence probability values: unsuitable habitats (0 ≤ *p* < 0.1, blue), lowly suitable habitats (0.1 ≤ *p* < 0.3, green), moderately suitable habitats (0.3 ≤ *p* < 0.5, yellow), and highly suitable habitats (0.5 ≤ *p* ≤ 1.0, red) [26,27]. The area of each suitable habitat level was then calculated.

Using a threshold probability of 0.1, the suitable habitats of *Gerbera piloselloides* were binary-classified into non-suitable habitats (assigned a value of 0) and suitable habitats (assigned a value of 1), regions transitioning from absence to presence (0→1) were classified as newly expanded suitable habitats, regions transitioning from presence to absence (1→0) were classified as vanished suitable habitats, and regions remaining transitioning from presence to presence (1→1) were classified as retained suitable habitats [28].

Grounded in the binary maps, the SDMtoolbox was used to calculate changes in the centroid positions of the potentially suitable habitats for *Gerbera piloselloides* over different time periods; the overall shift trends of the centroids were analyzed to reflect the impact of environmental changes on its distribution over time [29].

## 3. Results

### 3.1. Results of Model Optimization and Accuracy Evaluation

**Figure 4:**
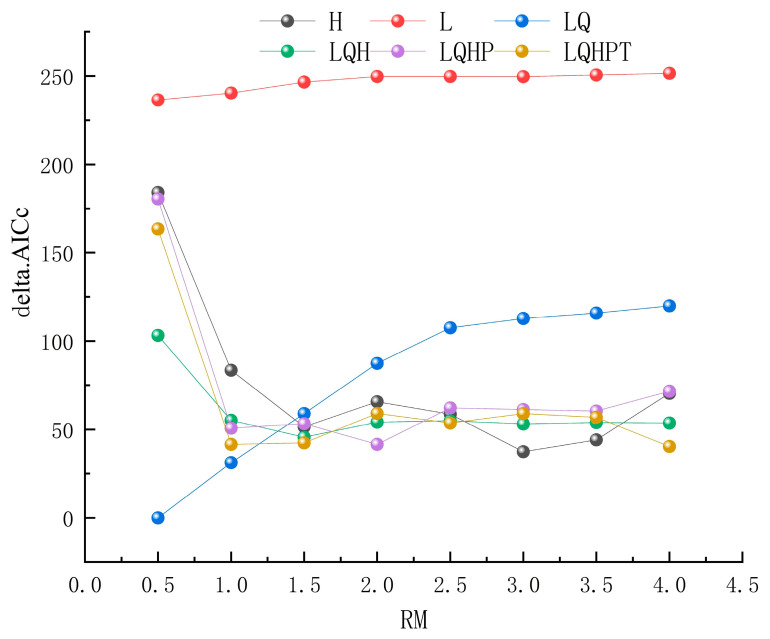
Optimization results of the MaxEnt model.

Parameter tuning using ENMeval showed that setting the regularization multiplier to 0.5 and selecting linear and quadratic features (LQ) minimized the AICc score (delta.AICc = 0); in comparison, the default MaxEnt settings (FC = LQHP, RM = 1) yielded a significantly higher delta.AICc (50.68) (Figure 4). Consequently, the parameters RM = 0.5 and FC = LQ were determined to be the optimal configurations. The model demonstrated strong predictive performance, with an average training AUC of 0.930 (Figure S1).

### 3.2. Major Environmental Factors Affecting Gerbera piloselloides Distribution

**Figure 5:**
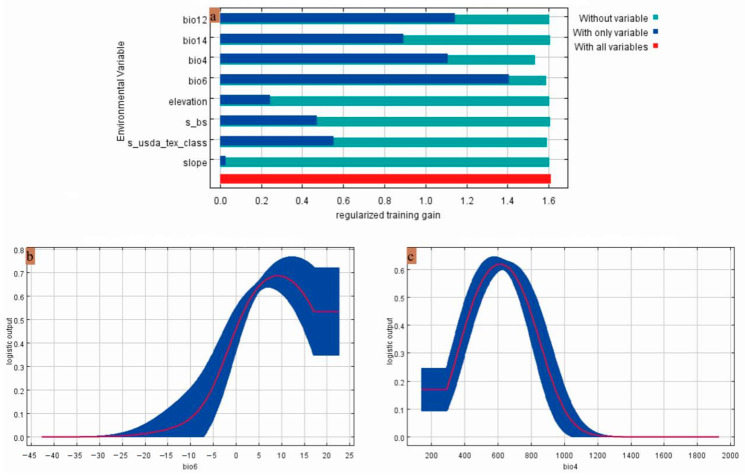
(**a**) Results of the jackknife test of factor importance for *Gerbera piloselloides*; (**b**) response curves of the bio6; (**c**) response curves of the bio4.

The analysis of environmental factor percent contribution and permutation importance revealed the bio6 (minimum temperature of the coldest month) and the bio4 (temperature seasonality) as the primary determinants of *Gerbera piloselloides* distribution, representing 79.3% of contribution and 90.5% of permutation importance (Table 2). The jackknife test results showed that among all environmental variables, the bio6 exhibited the highest gain value when used alone, suggesting that it provides the most significant independent information; in contrast, omitting the bio4 led to the largest decrease in gain, demonstrating that this factor contributes the most distinct information compared to the other variables (Figure 5a). In conclusion, the primary environmental factors influencing the potential distribution of *Gerbera piloselloides* are the bio6 and the bio4.

The occurrence probability of *Gerbera piloselloides* varied with environmental conditions (Figure 5b,c), and the key influencing ranges are summarized in Table 2. In highly suitable habitats, the bio6 ranged from 0.88 to 22.58 °C, and the bio4 ranged from 461.54 to 763.9. In the most suitable habitat, the bio6 was 9.24 °C, and the bio4 was 613.66.

### 3.3. Existing Potential Distribution of Gerbera piloselloides

**Figure 6:**
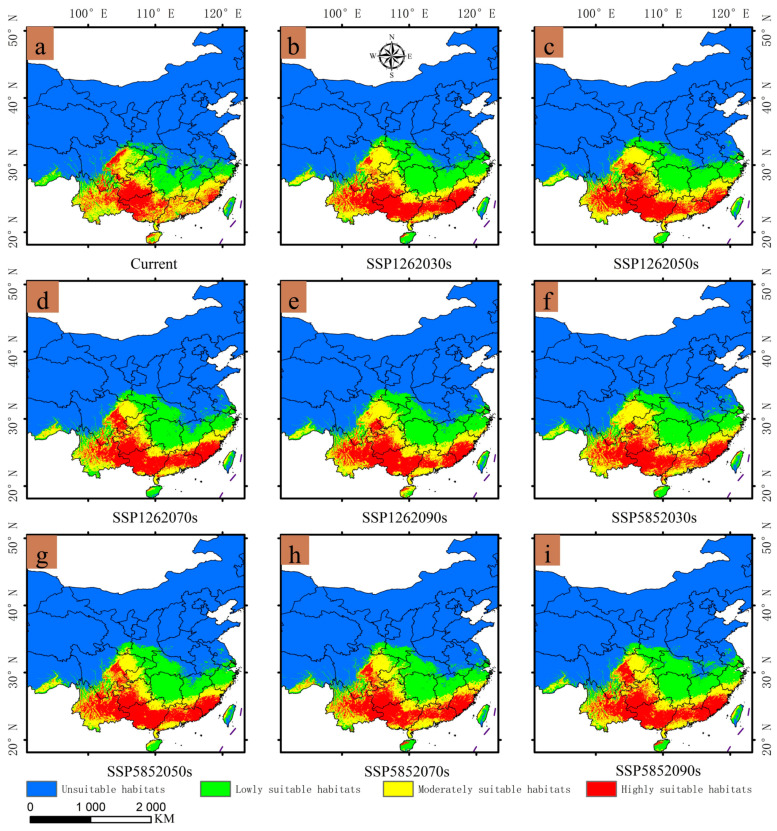
Potential distribution of *Gerbera piloselloides* under current and future climate projections: (**a**) Suitable habitats for *Gerbera piloselloides* in China under Current scenario; (**b**) SSP1262030s; (**c**) SSP1262050s; (**d**) SSP1262070s; (**e**) SSP1262090s; (**f**) SSP5852030s; (**g**) SSP5852050s; (**h**) SSP5852070s; (**i**) SSP5852090s.

As shown in Figure 6a, highly suitable habitats were concentrated in southern Guizhou, northern Yunnan, the majority of Guangxi, central Sichuan, eastern Fujian, Hainan, and certain regions of Guangdong. Moderately suitable habitats include eastern Sichuan, western Chongqing, northern Guizhou, western Fujian, southern Zhejiang, southern Jiangxi, western Taiwan, Hainan, most of Guangdong, extensive parts of Yunnan, and some areas of Xizang Autonomous Region (Xizang). In addition, habitats with a low level of suitability were mainly located in northern Zhejiang, northern Jiangxi, much of Hunan, and western Hubei. The predicted suitable habitats for *Gerbera piloselloides* covered all of its known distribution points, and the simulation results closely match the actual distribution. This consistency further validates the accuracy of the predictions in this study [30].

### 3.4. Expected Distribution Changes in Gerbera piloselloides in the Future

**Figure 7:**
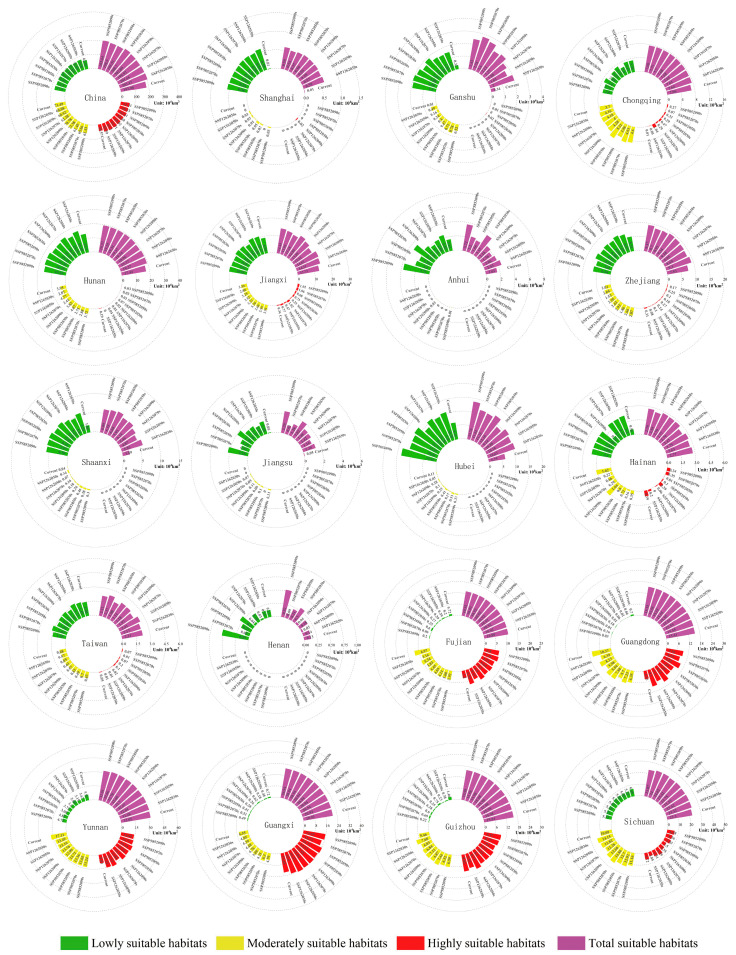
Areal changes in *Gerbera piloselloides* under multiple climate futures.

From Figure 6b–i, it can be seen that under different future climate scenarios, the suitable habitats for *Gerbera piloselloides* are primarily distributed south of the Qinling-Huaihe line in China, with expansions to the north and west. However, the number of suitable habitats declines in the border areas of the provinces of Jiangxi, Anhui, Hunan, and Hubei. As shown in Figure 7 and Figure S2, the overall suitable habitat area expanded, particularly in Shanghai, Gansu, Chongqing, Hunan, Jiangxi, Anhui, Zhejiang, Shaanxi, and Jiangsu. Areas of highly suitable habitats increased in Fujian, Guangdong, Yunnan, and Guangxi but decreased in Hainan, Guizhou, and Sichuan. Moderately suitable habitat areas expanded in Chongqing, Guizhou, Sichuan, and Shanghai but contracted in Fujian, Guangdong, Yunnan, Guangxi, and Hainan. In addition, habitat areas with low suitability increased in Shanghai, Hainan, Hubei, Shaanxi, Hunan, Jiangsu, Zhejiang, Taiwan, Jiangxi, and Gansu but decreased in Guizhou, Yunnan, Fujian, Guangdong, Sichuan, and Chongqing.

### 3.5. Migration Routes of the Suitable Habitat Centroid for Gerbera piloselloides

**Figure 8:**
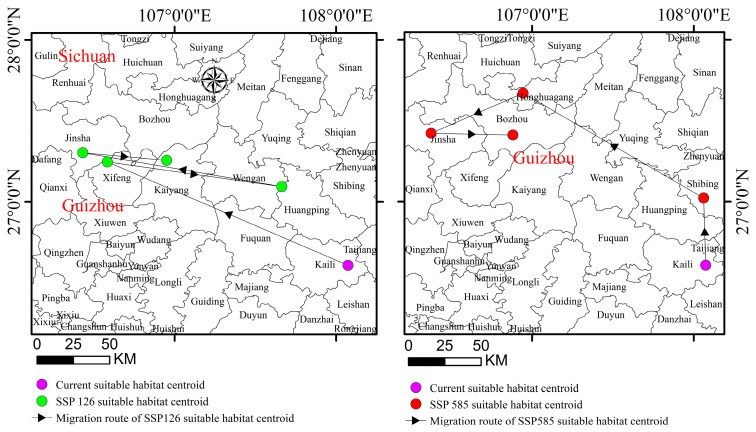
Migration routes of the suitable habitat centroid for *Gerbera piloselloides*.

The results presented in Figure 8 show that the current suitable habitat center for *Gerbera piloselloides* is situated in Kaili City (108.4° E, 26.36° N) within Guizhou province. Under the SSP126 climate scenario, the projections showed the following shifts: By the 2030s, the center will move to Xifeng County (106.35° E, 27.14° N), covering a distance of 164.0 km. By the 2050s, it will shift to Weng’an County (107.39° E, 27.5° N), spanning 108.8 km. By the 2070s, it will relocate to Jinsha County (106.25° E, 27.18° N), moving 124.2 km. By the 2090s, it will settle in Kaiyang County (106.57° E, 27.15° N), with a displacement of 51.7 km. Under the SSP585 scenario, the following changes were predicted: In the 2030s, the center will shift to Shibing County (108.3° E, 27.1° N), migrating 46.2 km. By the 2050s, it will move to Honghuagang District (106.56° E, 27.40° N), covering 131.8 km. In the 2070s, it will transition to Jinsha County (106.22° E, 27.25° N), spanning 62.5 km. By the 2090s, it will adjust to Bozhou District (106.52° E, 27.24° N), shifting 49.9 km. The findings suggest that, under future climate conditions, the centroid of the suitable habitats for *Gerbera piloselloides* will generally shift westward and northward. Notably, as greenhouse gas emission concentrations increased, the migration distance tended to expand, and the direction of migration gradually shifted northward.

## 4. Discussion

### 4.1. Environmental Determinants of Gerbera piloselloides Distribution: Temperature Constraints and the Qinling–Huaihe Divide

The proliferation and spatial distribution of plant species are shaped by a variety of environmental variables. Findings from the MaxEnt model suggest that the bio6 and bio4 are significant environmental determinants influencing the potential distribution of *Gerbera piloselloides*. Cold stress significantly restricts plant distribution and adversely affects their physiological processes, often resulting in lower agricultural productivity, deteriorated crop quality, and even mortality under extreme conditions [31]. Based on our findings, *Gerbera piloselloides* predominantly occurs in areas south of the Qinling–Huaihe line, a critical climatic boundary marking the January 0 °C isotherm and separating northern and southern China [32]. Predictive modeling indicates that the minimum temperature of the coldest month (bio6) in highly suitable habitats for this species varies between 0.88 and 22.58 °C. In addition, temperature seasonality (bio4), reflecting annual thermal variation in temperate zones, plays a key role in shaping climatic conditions [33]. In the eastern section of China’s north–south dividing zone, the Huaihe River Basin experiences abrupt transitions between cold and warm conditions, with extreme minimum temperatures reaching −24.1 °C, making it a frost-prone area with a historical frequency of 30–40%; periodic frost events have repeatedly caused significant damage to citrus cultivation areas in the Yangtze River Basin south of the Huaihe River [34,35]. In the western section of the north–south dividing zone, the Qinling Mountains block cold air from the north in winter, creating a favorable microclimate for citrus overwintering; this unique local microclimate has made Hanzhong, located at the southern base of the Qinling Mountain range, the northernmost citrus-growing region in China [36,37]. The variation in seasonal temperature fluctuations across the Qinling–Huaihe line likely contributes to the observed distribution pattern of *Gerbera piloselloides*. In eastern regions, the species’ northern limit predominantly occurs south of the Yangtze River; in comparison, western populations extend further north to the Qinling Mountains’ southern slopes. Model predictions indicate that the bio4 in highly suitable habitats for *Gerbera piloselloides* ranges from 461.54 to 763.96.

### 4.2. Divergent Responses to Climate Change: Habitat Expansion in Northern Limits vs. Contraction in the Yangtze River Basin

The IPCC’s AR6 indicates with high confidence that anthropogenic warming will reach the 1.5 °C threshold above pre-industrial levels during the 2021–2040 timeframe, accompanied by intensified regional precipitation variability [38]. The findings of this study suggest that while *Gerbera piloselloides* will predominantly retain its suitable habitats south of China’s Qinling–Huaihe line, its distribution is likely to shift toward higher latitudes and elevations. Under escalating greenhouse gas concentrations, the species’ range is anticipated to expand, particularly along its northern distributional limits. However, climate change is also exerting divergent effects on *Gerbera piloselloides* distribution. A contraction is observed in the junction areas of Jiangxi, Anhui, Hunan, and Hubei provinces, potentially linked to heightened flood risks in the Yangtze River Basin due to climate-driven precipitation increases [39]. Notably, Fujian, Guangdong, Yunnan, and Guangxi are expected to experience a reduction in low-to-moderately suitable habitats but an expansion of highly suitable habitats. In contrast, in Guizhou and Sichuan, the number of highly suitable habitats may contract; in comparison, the number of habitats of low suitability may increase. This disparity could arise because warmer, wetter conditions favor *Gerbera piloselloides* in southeastern provinces; in comparison, the Yunnan–Guizhou quasi-stationary front elevates the risk of winter frost damage in southwestern regions despite overall precipitation gains [40].

### 4.3. Significance and Limitations of the Study

*Gerbera piloselloides* and *Gerbera jamesonii* (African daisy) belong to the same genus, *Gerbera*, within the Asteraceae family. African daisies are not cold-tolerant, and their growth declines in low-temperature environments. They require temperature and humidity control during winter; to ensure their survival, nighttime temperatures should not fall below 4 °C, as such conditions can halt growth and cause frost damage. Extended exposure to temperatures below 0 °C can lead to plant death [41,42,43]. Our research group has previously conducted artificial cultivation studies of *Gerbera piloselloides* in Guizhou province. The average winter temperature in Guizhou ranges from 4 to 6 °C, with occasional extreme cold weather accompanied by the temperature dropping to −6 to −9 °C; although such conditions are short-lived, failure to take appropriate measures can result in frost damage and significant production losses for African daisies [43]. In this study, we found that the bio6 exerts a considerable influence on the distribution patterns of *Gerbera piloselloides,* with a percent contribution of 59.6% and a permutation importance of 50.8%. The bio6 in highly suitable habitats ranges from 0.88 to 22.58 °C, with the most suitable habitat observed at 9.24 °C.

The above findings suggest that the disease-related issues encountered in the artificial cultivation of *Gerbera piloselloides* may be similar to the cold intolerance observed in African daisies. Introducing medicinal plants to a region without comprehensive consideration of relevant environmental factors can lead to issues related to pests and disease [44]. The highly suitable habitats for *Gerbera piloselloides* in Fujian, Guangdong, Yunnan, and Guangxi remain stable over time, with them even exhibiting a gradual increase. This stability suggests that these regions are promising locations for artificial introduction and cultivation experiments. Delineating species distribution areas is crucial for conservation decision-making [45]. The border areas of the provinces of Jiangxi, Anhui, Hunan, and Hubei are experiencing a reduction in suitable habitats compared to the current period. It is recommended that efforts be made to strengthen the collection of germplasm resources of *Gerbera piloselloides* in these regions to preserve its genetic diversity to the greatest extent possible. The distribution of *Gerbera piloselloides* is shaped not only by abiotic factors (e.g., climate, soil, and topography) but is also potentially impacted by human activities (e.g., Human Footprint Index [HFI] and Human Influence Index [HII]). However, given the current absence of reliable future projections for human activity variables [46,47], our species distribution models did not explicitly incorporate these anthropogenic components. The authors of subsequent studies could enhance model accuracy by integrating newly available human activity projection datasets when such data become accessible.

## 5. Conclusions

The findings presented in this study provide definitive answers to the four research objectives outlined in the Introduction: (1) Current suitable range: Our models identify the core distribution of *Gerbera piloselloides* south of the Qinling–Huaihe line, with highly suitable habitats concentrated in southern Guizhou, northern Yunnan, the majority of Guangxi, central Sichuan, eastern Fujian, Hainan, and certain regions of Guangdong. (2) Future distribution shifts: Under climate change scenarios, suitable habitats will extend in a northerly and westerly direction; in comparison, habitat contraction is expected near the borders of the provinces of Jiangxi, Anhui, Hunan, and Hubei. Highly suitable habitats are expanding in Fujian, Guangdong, Yunnan, and Guangxi but declining in Guizhou, Sichuan, and Hainan. (3) Key limiting factors: The minimum temperature of the coldest month (bio6) is the dominant constraint, with temperature seasonality (bio4) serving as a secondary regulator of distribution. The species exhibits optimal distribution when the bio6 ranges from 0.88 to 22.58 °C and the bio4 ranges from 461.54 to 763.9. (4) Based on habitat stability and suitability thresholds, we recommend the following zones for cultivation: The stable, highly suitable habitats include Fujian, Guangdong, Yunnan, and Guangxi, particularly areas maintaining suitability across all climate scenarios. Conservation priority areas: These areas include the borders of Jiangxi, Anhui, Hunan, and Hubei provinces. These recommendations integrate both current distribution patterns and projected climate change impacts, providing a research-based framework for the species’ sustainable utilization and protection.

## Figures and Tables

**Table 1:** Environmental factors used for modeling.

Factor Name	Description	Percent Contribution
bio12	Annual precipitation	37.8
bio6	Minimum temperature of the coldest month	19.8
bio14	Precipitation of the driest month	14.1
bio4	Temperature seasonality	4.9
Elevation	Elevation	3.2
s_usda_tex_class	Subsoil USDA texture classification	1.3
Slope	Slope	1.2
s_bs	Subsoil base saturation	1

**Table 2:** Range of key environmental factors in highly suitable habitats for *Gerbera piloselloides*.

Factor Name	Description	Percent Contribution	Permutation Importance	Highly Suitable Habitat	Most Suitable Habitat	Unit
bio6	Minimum temperature of the coldest month	59.6	50.8	0.88–22.58	9.24	°C
bio4	Temperature seasonality	19.7	39.7	461.54–763.96	613.66	

## Data Availability

The data are included in the article. For the data provided in this study, see Section 2.1 and Section 2.2 in the text.

## Supplementary Materials

The following supporting information can be downloaded at https://www.mdpi.com/article/10.3390/biology14070769/s1. Table S1. 58 environment variables; Figure S1. Receiver operating characteristic curve; Figure S2. Areal changes in *Gerbera piloselloides* under multiple climate futures.
