# Supplementary material for: Assessing Suitable Habitats for Gerbera piloselloides (L.)Cass. in China Using an Optimized MaxEnt Model and Key Environmental Drivers
Source: Biology (Basel). 2025 Jun 26;14(7):769. doi: 10.3390/biology14070769 (PMC12292698; doi:10.3390/biology14070769)
Supplement: Supplementary file 1 [file biology-14-00769-s001.zip › Supplementary Materials.pdf]

Supplementary Materials

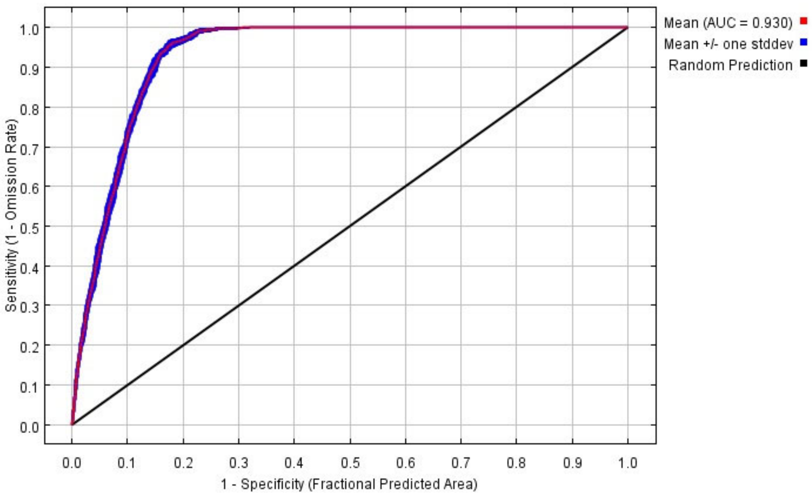

Figure S1. Receiver operating characteristic curve.

Table S1. 58 environment variables.

Variable		Description	Units
bioclimatic variables	bio1	Annual mean temperature	℃
	bio2	Mean diurnal range	℃
	bio3	Isothermality	1
	bio4	Temperature seasonality	-

	bio5	Max temperature of warmest month	℃
	bio6	Minimum temperature of the coldest month	℃
	bio7	Temperature Annual Range	℃
	bio8	Mean temperature of wettest quarter	℃
	bio9	Mean temperature of driest quarter	℃
	bio10	Mean temperature of warmest quarter	℃
	bio11	Mean temperature of coldest quarter	℃
	bio12	Annual precipitation	mm
	bio13	Precipitation of wettest month	mm
	bio14	Precipitation of the driest month	mm
	bio15	Precipitation seasonality	-
	bio16	Precipitation of wettest quarter	mm
	bio17	Precipitation of driest quarter	mm
	bio18	Precipitation of warmest quarter	mm
	bio19	Precipitation of coldest quarter	mm
	t_usda_tex_class	Topsoil USDA texture classification	name
	s_cec_clay	Subsoil CEC (clay)	cmol/kg
	s_usda_tex_class	Subsoil USDA texture classification	name
	t_gravel	Topsoil Gravel Content	%vol.
	drainage	Drainage class	name
Soil variables	s_bs	Subsoil base saturation	%
	s_cec_soil	Subsoil CEC (soil)	cmol/kg
	s_gravel	Suboil Gravel Content	%vol.
	awc_class	Soil available water content	-
	s_bulk_den	S_bULK_DEN	-
	s_caco3	Subsoil calcium carbonate	% wt.

s_clay	Subsoil clay fraction	% wt.
s_ph_h2o	Subsoil pH（H2O）	-log（H^+）
t_bs	Topsoil base saturation	name
t_caco3	Topsoil calcium carbonate	% wt.
t_cec_clay	Topsoil CEC (clay)	cmol/kg
t_sand	Topsoil sand fraction	% wt.
s_caso4	Subsoil gypsum	% wt.
s_ece	Subsoil salinity (Elco)	dS/m
s_esp	Subsoil sodicity (ESP)	%
s_oc	Subsoil organic carbon	% wt.
s_ref_bulk_density	Subsoil reference bulk density	kg/dm3
s_sand	Subsoil sand fraction	% wt.
s_silt	Subsoil silt fraction	% wt.
s_teb	Subsoil TEb	cmol/kg
t_caso4	Topsoil gypsum	% wt.
t_cec_soil	Topsoil CEC (soil)	cmol/kg
t_clay	Topsoil clay fraction	% wt.
t_ece	Topsoil salinity (Elco)	dS/m
t_esp	Topsoil sodicity (ESP)	%
t_oc	Topsoil organic carbon	% wt.
t_ph_h2o	Topsoil pH（H2O）	-log（H^+）
t_ref_bulk_density	Topsoil reference bulk density	kg/dm3
t_silt	Topsoil silt fraction	% wt.
t_teb	Topsoil TEb	cmol/kg

	t_texture	Topsoil texture	name
Topographic variables	elevation	Elevation	m
	slope	Slope	°
	aspect	Aspect	°
